# Spatiotemporal Thermal Modulation and Patterning using a Programmable 1024 Element Microheater Array

**DOI:** 10.1002/advs.77563

**Published:** 2026-09-11

**Authors:** Rahul Goyal, Jang‐Hwan Han, Sadaf Pashapour, Peer Fischer

**Affiliations:** ^1^ Institute for Molecular Systems Engineering and Advanced Materials Heidelberg University Heidelberg Germany; ^2^ Max Planck Institute for Medical Research Heidelberg Germany; ^3^ Center for Nanomedicine Institute for Basic Science (IBS) Seoul Republic of Korea; ^4^ Department of Nano Biomedical Engineering (NanoBME), Advanced Science Institute Yonsei University Seoul Republic of Korea

**Keywords:** electronics, fabrication, microheater, multiplexer, patterning, printing, thermal

## Abstract

Programmable microheater arrays are essential for a variety of applications including gas sensing, microfluidic lab‐on‐a‐chip devices, 3D printers, and biosensors that rely on DNA amplification. Increasing the density and number of heating elements directly correlates with the precision with which spatiotemporal heat profiles can be delivered. However, large arrays have thus far not been realized. One challenge is that as the number of elements in an array increases, the complexity of connecting them grows. Here, we show that row‐column addressing provides a promising architecture for the efficient operation of a large microheater array. We introduce a programmable 32×32 microheater array consisting of individually addressable robust platinum (Pt)‐based Joule heating elements – each smaller than 300μm. We show that combining high‐voltage multiplexed electronics and sequential addressing controlled by a high‐frequency clock, allows the independent operation of the 1024 microheater elements. We demonstrate the generation of heat images and the patterning of metallic structures formed from the liquid metal Gallium. Our work demonstrates new capabilities for on‐chip thermal devices, and opens the possibility to realize novel heat‐controlled micro actuation systems.

## Graphical abstract

Programmable microheater arrays are essential for a variety of applications. However, large arrays have thus far not been realized. We introduce a programmable 32×32 microheater array consisting of heating elements – each smaller than 300 μm. We demonstrate the generation of heat images and the patterning of metallic structures. Our work demonstrates new capabilities for novel on‐chip heat‐controlled micro actuation systems.

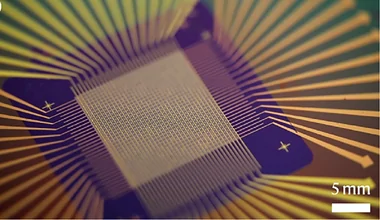

## 1 Introduction

Microheater elements with programmable heat generation and spatial control have potential applications in a range of fields [1, 2], including haptic displays [3, 4, 5], gas sensors [6, 7], actuation and robotics [8, 9, 10], 3D printing [11], electronic skin [12], and thermal encoding [13, 14, 15]. The specific response‐time requirements vary considerably across these applications, and the present work focuses primarily on demonstrating scalable spatial and temporal thermal control rather than application‐specific high‐speed operation. The most conventional methodology utilizes pixelated heating elements integrated with direct wire addressing circuits [16]. This approach can in principle generate complex thermal patterns, but has limited scalability. Recently, advanced materials such as multi‐layer carbon‐fiber‐reinforced polymers, carbon nanotubes (CNTs) [17, 18], Fe/Ni/Co alloy (Invar) [19] have also been incorporated in heater designs, thereby broadening potential applications. Still, demonstrations have been limited to directly‐wired single heating elements [20] or to small array sizes [21]. The scale‐up to larger multi‐pixel arrays presents a major challenge, demanding uniform electrical characteristics of the material across all the elements of an array [3, 4, 22], as well as a scalable architecture.

Large numbers of fine, closely spaced heating elements promise the generation of sophisticated heat patterns and potentially steep thermal gradients. However, as the feature size decreases it becomes progressively more difficult to generate sufficient heat, since the resistance drops with shorter conductor lines, and a significant difference in the impedance of the microheater cell and the interconnected input power lines is needed. Moreover, it becomes challenging to address large arrays, as wiring large number of heaters becomes challenging. For instance, wiring each element separately becomes impractical. Herein, we show how high‐speed row/column addressing can be used to overcome these challenges and how this enables the selective activation of any individual elements within the array. We thereby demonstrate the fabrication, control, and operation of what, to the best of our knowledge is the largest microheater array to date. A detailed comparison between the current work and previously reported microheater arrays is presented in the Table S1. We use our array to show how the generated heat patterns can be used to form structures made from liquid metals.

**FIGURE 1:**
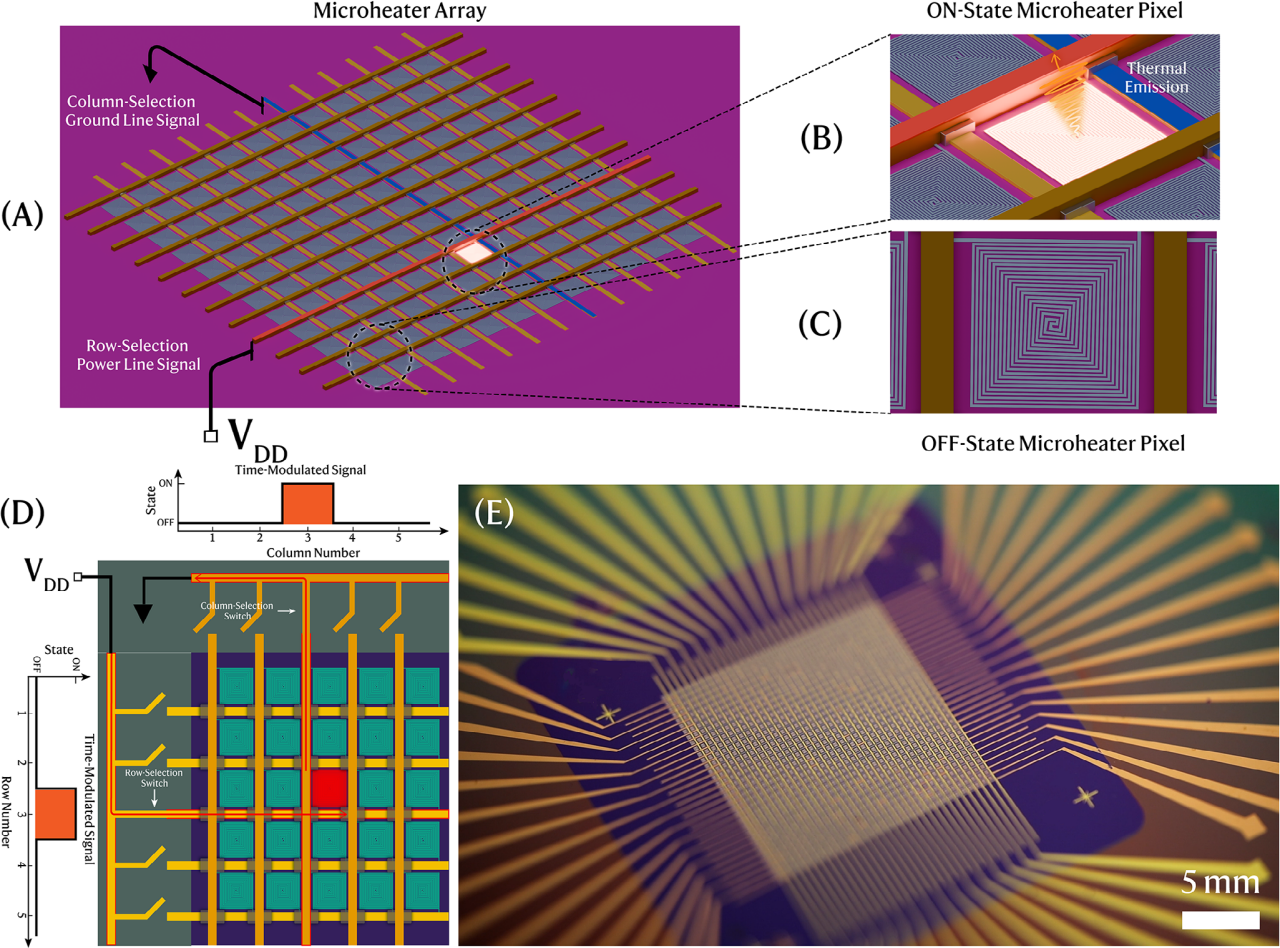
Presentation of fully integrated microheater array for spatial thermal modulation. (A) Schematics of the process of thermal emission in a microheater array with a row‐column addressing architecture. (B) The connection of power and ground lines determines the operational state of the microheater pixel, specifically enabling it to function in the (B) ON‐State, while ensuring that all other pixels remain in (C) OFF‐State. (D) The row‐ and column‐selection switches are externally operated through a time‐modulated digital signal that controls the state of pixel. (E) Optical photograph of 2D microheater array fabricated on a three‐inch Si/SiO_2_ (300nm) wet thermal oxide silicon wafer. The wafer contains a total of 1024 double‐spiral square layout‐shaped microheaters.

Our microheater array consists of 1024 elements, arranged in a 32×32 closely spaced grid, where each heater has a dimension of 297.5μm. A schematic with the design and operating concept is illustrated in Figure 1A. Each microheater can be independently addressed to generate spatiotemporal thermal profiles (see Movie S4 for further details). We adopt a double‐spiral square microheater geometry (Figure 1B,C), which provides efficient high‐temperature operation and localizes the heat for spatiotemporal thermal control. The modulation is performed through multiplexer electronics. The array has 64 independent control channels, with 32 rows and columns, each equipped with an independent switch control circuit. This permits the direction and polarity of the voltage signal to be controlled, thereby achieving a time‐modulated powering of individual microheater elements. Figure 1D depicts the schematic circuit diagram of a 5×5 array, consisting of 25 resisting elements for joule heating operations. The entire 32×32 microheater array is seen in Figure 1E.

## 2 Design and Characterization of the Device

**FIGURE 2:**
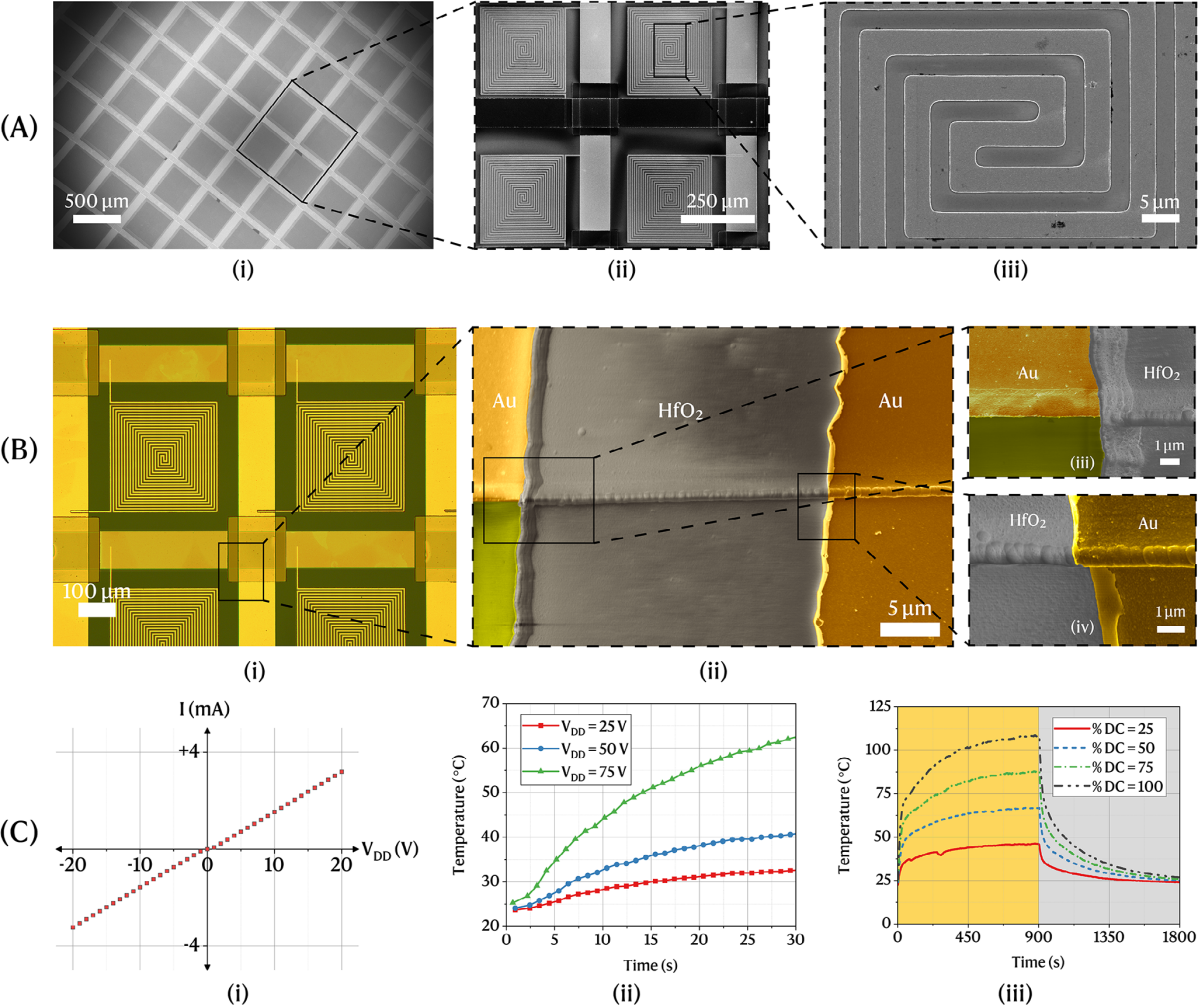
Structure of the microheater array and electrical characterization. (A) Scanning electron microscopy images of the layout of microheater array. (i) Input and output terminals are connected to the square‐layout shaped microheaters for multiplexed operation. (ii) Enlarged image of inset, showing a 2×2 subset. (iii) Magnified double‐spiral structure of a microheater element. (B) False‐colored scanning electron microscopy image of the microheater array where ${\mathrm{HfO}}_{2}$, Au, and ${\mathrm{SiO}}_{2}$ are shown in grey, yellow and green colors, respectively. (i) Layout of the microheater array connected through row‐column architecture. (ii) The overlapped region between input/output bus lines and crossover isolated area of the device. (iii) ${\mathrm{HfO}}_{2}$ layer isolates the input busline from the output busline. (iv) Output busline patterned over the ${\mathrm{HfO}}_{2}$ to establish electrical connection with the microheater elements. (C) Experimental measurements for microheater element. (i) Electrical characteristic curve of an element when $V_{DD}\leq 20\;V$. (ii) The effect of DC voltage ($V_{DD}$) on the heating performance under 30s of continuous operation. (iii) The effect of duty cycle (%DC) on the heating and cooling performance of the element under 75V AC square wave signal.

An overview of the microheater array and the scheme used to address elements together with images of the device is shown in Figure 1. Each microheater element in the 32×32 square‐array has a side length of 297.5μm and consists of a square‐shaped double spiral Pt conductor line. We chose Pt due to its low thermal expansion coefficient ($∼8.9\times 10^{-6}\;K^{-1}$) and high resistivity ($∼10.1\times 10^{-8}\Omega m$). Moreover, Pt provides advantage over other materials which necessitate highly sophisticated fabrication processes that includes electrical interfacing of the advanced materials which substantially complicates the overall system [19, 23, 24, 25, 26, 27]. The center‐to‐center distance between heater elements is 500μm. The entire array measures 15.5mm×15.5mm, as shown in Figure 1E. Each element has in total 40 variable radius turns with a minimum feature size of approximately 2μm. The false‐color scanning electron microscopy (SEM) images of the device and the corresponding crossover junction are shown in Figure 2B(i–iii). Since row/column addressing is used (see below for details), the crossing conductor lines are isolated from each other by a 1μm thick HfO_2_ layer (see Methods for details). The HfO_2_ layer can be observed in Figure 2B(ii), (iii). The SEM image shows the top Au bus line fabricated over the HfO_2_ dielectric layer. The experimentally measured current‐voltage response of a single microheater element is shown in Figure 2C(i), and confirms the purely resistive impedance in the operating range (peak‐to‐peak amplitude of the AC voltage signal is kept constant at max. 120V). The statistical distribution of the resistance of individual Pt microheater elements as well as thermoresistive response across the investigated temperature range are shown in Figure S7. The temperature coefficient of resistance (TCR) was determined by fitting the linear relation $R=R_{0}\left(1+\alpha \Delta T\right)$ to the measured data, yielding α = 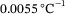. The experimental results of the temperature recorded at different rates of the driving voltage are presented in Figure S1, and confirm that the purely resistive heating characteristics are independent of the pulse rate.

Contacting individual heater elements with direct electrical connections is not scalable and next to impossible for a dense microheater array. Instead, we activate individual elements by row/column addressing (see Movie S4 for further details) and use a scheme where the heating elements are supplied with periodic square‐wave voltage pulses. This permits the control of the target temperature via the percentage duty cycle (%DC), which corresponds to the fractional ON time ($T_{ON}$) with respect to a maximal signal time of (10ms), and the total number of pulses applied. The temperature evolution recorded under ambient conditions from a single heating element in response to a square‐wave pulsating signal with a constant frequency of 100Hz and increasing voltages is depicted in Figure 2C(ii). The steady state temperature profiles as a function of the applied voltages are shown in Figure S1. Next, we demonstrate the temperature profiles when a constant peak voltage of 75V is applied and the duty cycle is varied. The ON time of 2.5ms, 5.0ms, 7.5ms, and 10ms, correspond respectively to %DC of 25%, 50%, 75%, and 100%. The results are shown in Figure 2C(iii). The temperature of a heating element will henceforth be controlled by varying the %DC ($T_{ON}$) while keeping the voltage $V_{DD}\leq 75\;V$.

## 3 Multiplexed Operation of Microheater Array

**FIGURE 3:**
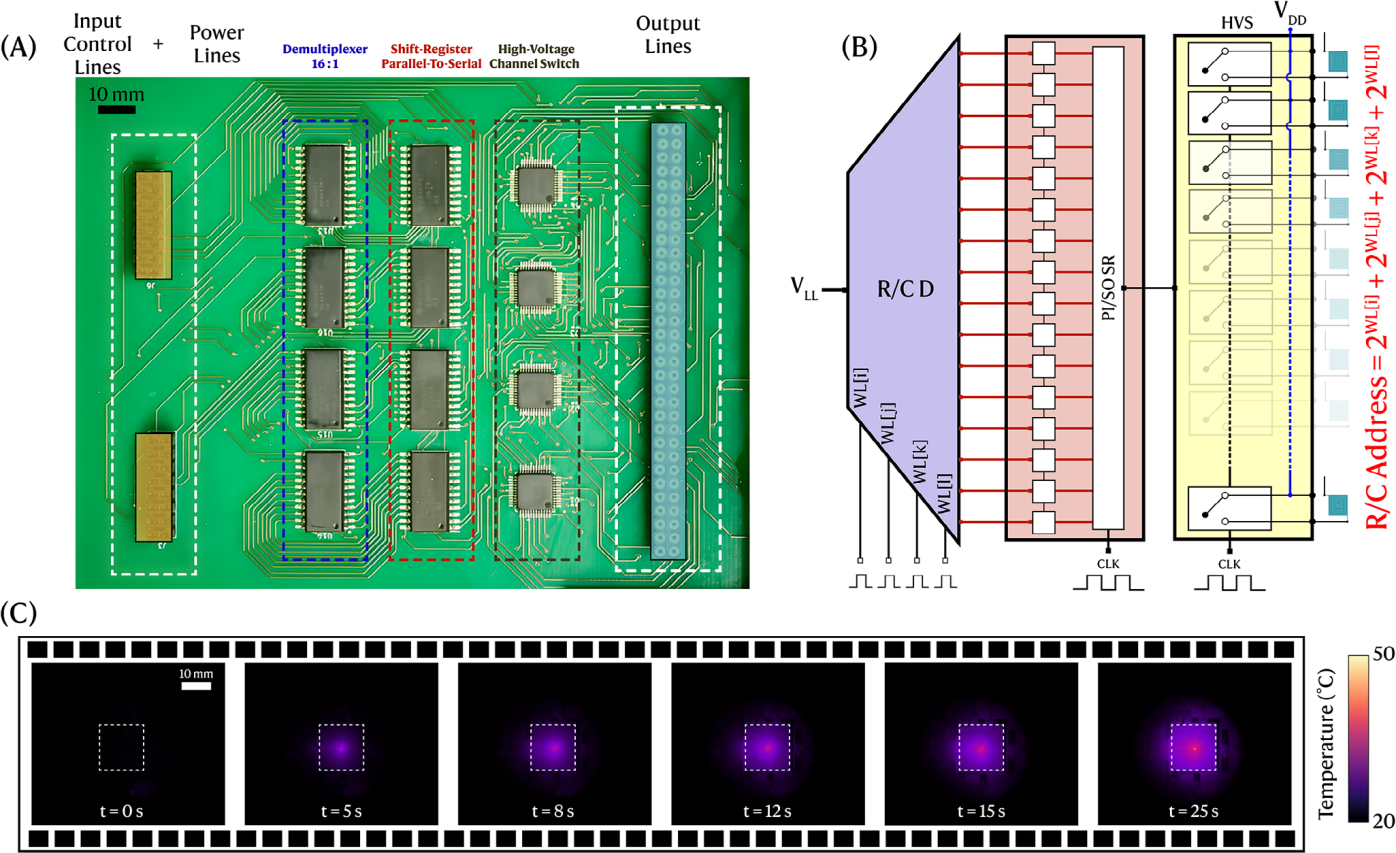
High‐speed multiplexing circuit layout used to address individual heater cells in the 32×32 array via a serial input driven by an external clock (CLK). (A) Schematic of the bus‐line addressing architecture with the associated bit‐transfer sequence. (B) Photograph of the fabricated printed circuit board implementing the corresponding row–column addressing scheme. (C) Infrared images of a single activated heater element at position (16, 16), with the temperature evolution over time as measured in air.

We now describe the row/column addressing architecture to achieve programmable control of the 32×32 pixels for a prototype microheater array. Figure 3 (A) shows the image of the printed circuit board that selects the respective row/column of the microheater array for multiplexing. The multiplexer board operates the 32 buslines at 5 bits. Figure 3B shows the signal diagram of the multiplexer board in conjunction with the high voltage switches. More information about the control architecture can be found in the Methods Section 8 as well as in the Note S2 and Figure S9. Infrared (IR) images captured from the operation of an individual element (16,16) at different time instants are shown in Figure 3C (see also Movie S1). The maximum temperature recorded by the IR camera after 25s of operation is 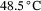, where a DC input voltage of 75V has been provided with a clock frequency of 1kHz and a pulse repetition rate of 250pulses/s. The instantaneous current per activated row is approximately 20mA. The corresponding instantaneous power ($P_{ON}$), with a driving voltage of 75V, is 1.5W (P = IV). However, since the microheaters are pulsed and the duty cycle regulates the ON/OFF state, the time‐averaged power ($P_{A}$) over a heating cycle is 0.75W. This value accounts for the fractional ON‐time and reflects the practical, average power consumption during sequential addressing. As the heaters are operated in a pulsed mode, the time‐averaged power is lower than the instantaneous value; it is given by $P_{A}=P_{ON}\times$ Duty Cycle. A detailed explanation of power calculations is presented in Note S1. When the temperature of a single microheater element in the array is kept at 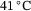, we observe that heat spreads to adjacent elements. The full width at half maximum (FWHM) of the temperature profile corresponds to approximately the width of four heating elements, as shown in Figure 3C. In what follows, multiple heating elements are addressed to form heat patterns.

## 4 Forming Spatial Heat Patterns by Spatially Selective Heating

**FIGURE 4:**
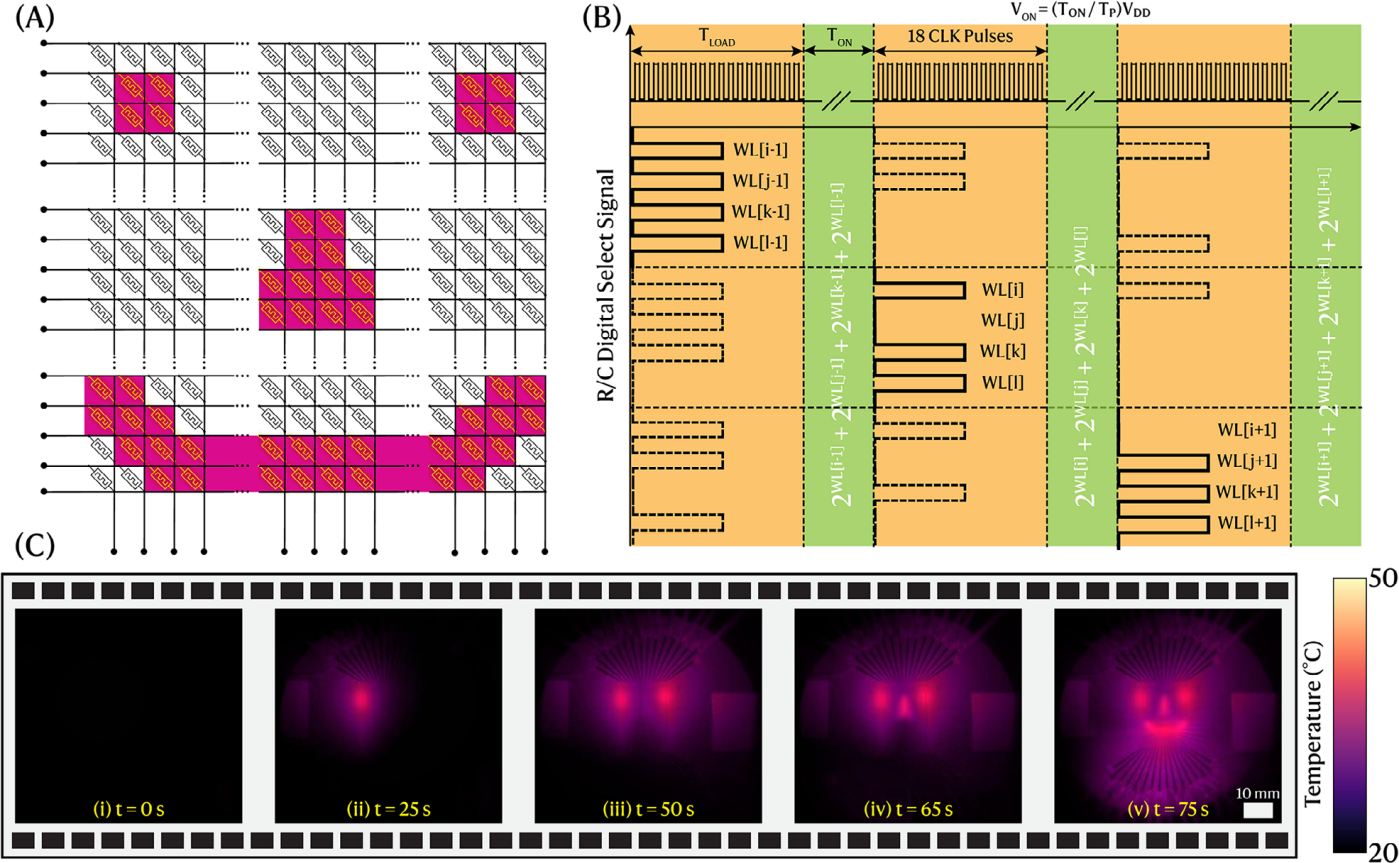
The demonstration of a 32×32 pixelated microheater array with row/column addressing for complex thermal pattern formation. (A) The circuit model for the pixelated microheater array to generate a complex thermal pattern. (B) The single cell can be activated by switching ON/ OFF the driving voltage line and ground signal line using an R/C digital select signal. The address of the activated pixel element is controlled through 4 bits (WL[i], WL[j], WL[k], and WL[l]) which are loaded in the high‐speed multiplexing circuitry using 18 CLK pulses. (C) The infrared images of a complex thermal pattern in the shape of “smiley face” displayed at various time instants; (i) t=0s, (ii) t=25s, (iii) t=50s, (iv) t=65s, and (v) t=75s.The minimum and maximum temperatures are 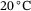 and 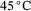, respectively.

We now describe how our device is used to form thermal images, as shown schematically in Figure 4. Specific elements in the array are addressed by loading a four‐bit word line which initiates the execution of the corresponding high voltage switch (HVS). For the depicted pattern, in total 18 clock (CLK) pulses, with a CLK signal at 25kHz and the $T_{ON}$ of 2ms was used. The times have been estimated based on the number of activated microheater elements and the target temperature. The elements are sequentially addressed using time‐division multiplexing as shown in Figure 4B. The control process is schematically shown in Figure 4B. The experimentally recorded sequence of infrared images corresponding to the thermal output of the heater array is depicted in Figure 4C (see also the Movie S2). The relatively low thermal diffusion to neighboring elements resulted in localized heating and a temperature difference between the activated and non‐activated microheater elements of at least 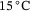, enabling the formation of distinct thermal patterns. The results for thermal crosstalk measured from all 1024 individually addressable microheater elements are shown in Figure S8.

## 5 Heat Patterning of Structures with Liquid Metals

**FIGURE 5:**
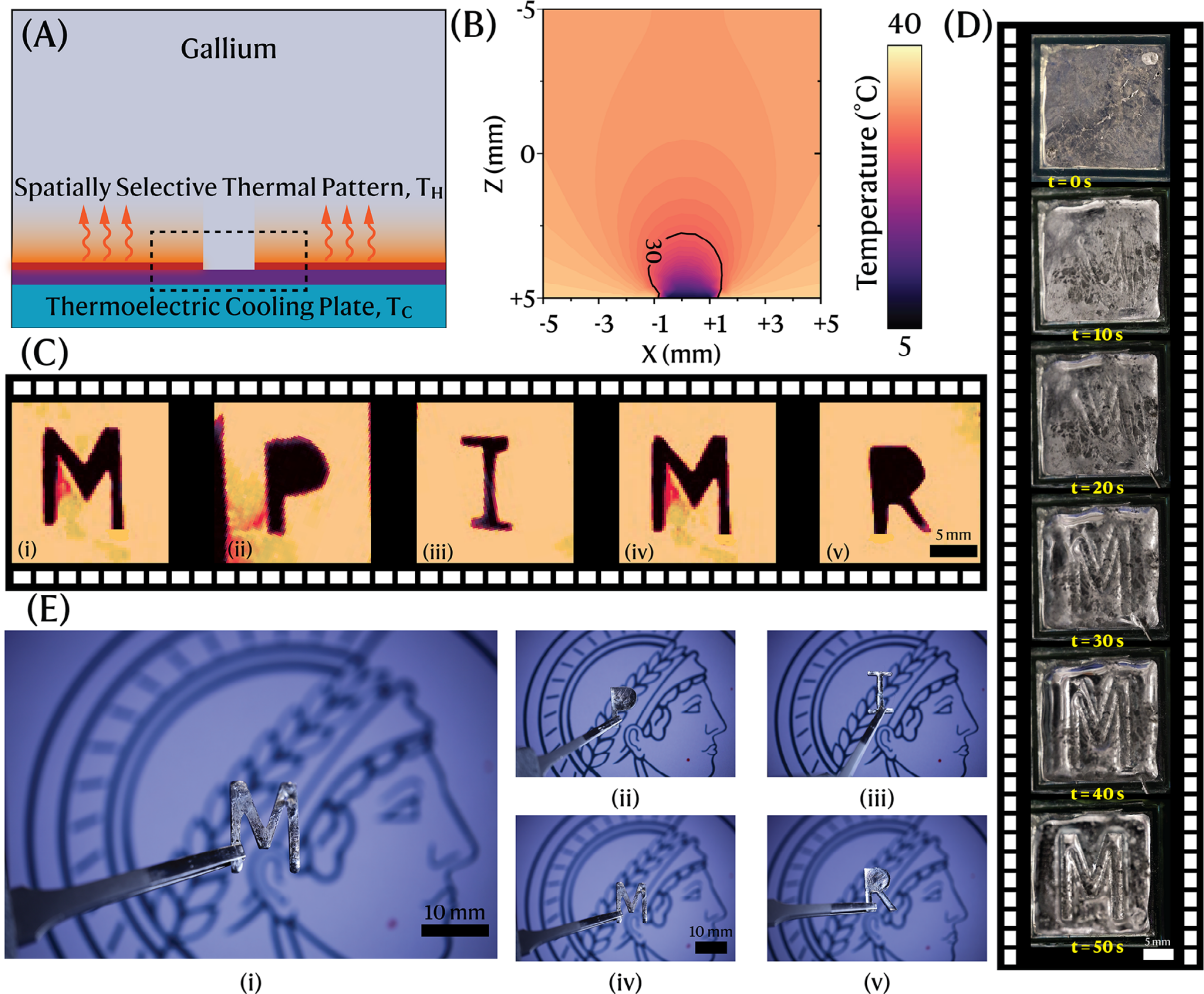
Thermal patterns formed in a liquid metal by the heater array. (A) Schematic illustration of the principle of pattern formation. The thermoelectric cooling plate at temperature $T_{C}$ is incorporated to ensure a stable base temperature, where the heater elements generate a temperature given by $T_{H}-T_{C}$. (B) The numerical results of the heating process simulated with a 2D model showing the surface distribution of the temperature at t=50s. (C) Sequential infrared images of the thermal patterns resembling the letters: “M,” “P,” “I,” “M,“ and “R.” The results at (i), (ii), (iii), (iv), and (v) show the surface distribution of the temperature for the letters. (D) The optical photographs of the “thermal printing” process at various time points. (E) Photographs of different structures of Gallium‐based liquid metal in the shape of the letters (i) “M,” (ii) “P,” (iii) “I,” (iv) “M,” and (v) “R”.

We demonstrate the operation of the programmable microheater array by forming structures in a liquid metal layer. Precise control over both the temperature and the spatial heat distribution are required to form the letters. Liquid metals are of interest for a number of applications, including flexible displays [28], strain sensors [29], antennae [30, 31], and biomedical sensors [32, 33]. Conventional additive manufacturing enables fast multidimensional fabrication of liquid metal structures with intricate designs for circuitry in flexible electronics [34]. However, liquid metals are challenging to handle due to their material properties. Their high surface tension and the formation of an oxide shell require that printers are customized [35, 36, 37] or that the rheological properties of the liquid metal are changed [38, 39, 40]. However, these changes may increase the complexity of the additive manufacturing process or deteriorate the electrical properties of the liquid metal. Here, we utilize the relatively sharp solid–liquid transition of Gallium together with the spatiotemporal modulation of the microheater array to pattern the Gallium via a subtractive process. The device is contacted to a thermoelectric cooling plate at a temperature T_C_ to set a base temperature. The Gallium is solid below $29.76^{\circ }$ C. The letters are formed by only heating those elements where the metal is to be removed, which is achieved by melting the Ga and removing the liquid Gallium. The pattering method is schematically shown in Figure 5A. The heat distribution along and extending away from the surface in the area indicated by the black dotted line of Figure 5A is shown in Figure 5B. The results are shown after 50s operation of the heater with T_H_ and T_C_ corresponding to 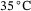 and 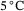, respectively. We generated Gallium in the shape of the letters “M,” “P,” “I,” “M,” and “R,” as shown in Figure 5C(i–v), respectively. The real‐time photographs of the patterning of Gallium captured at different time points are shown in Figure 5D (see also Movie S3). After the shape is formed, the remaining solid Gallium is detached from the surface using a tweezer. The resultant structures are shown in Figure 5E.

The Finite Element Method (FEM) simulation in Figure 5B was performed to investigate the local heat diffusion and thermal confinement associated with a single microheater element. The simulation predicts a strongly localized temperature rise at the heater location with a gradual temperature decay into the surrounding substrate. Experimentally, the IR thermal images exhibit a similar localized heating behavior, with the temperature confined primarily to the activated heater regions and a measured thermal spread of approximately four heater pitches at the FWHM. The qualitative agreement between simulation and experiment indicates that the FEM model adequately captures the dominant heat transport mechanisms governing thermal pattern formation in the microheater array.

## 6 Dynamic Thermal Actuation in Microfluidics Using PNIPAM

Poly(N‐isopropylacrylamide) (PNIPAM) is a thermoresponsive hydrogel extensively utilized in microfluidic applications due to its reversible volume‐phase transition at modest temperatures. It possesses a lower critical solution temperature (LCST), which occurs at approximately 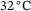 [41, 42]. Below the LCST, PNIPAM is in a hydrated swollen state. Beyond the LCST, the polymer network undergoes a rapid collapse, becoming hydrophobic and expelling water, resulting in a substantial reduction in volume [43]. This transition is driven by a shift from hydrogen bonding to hydrophobic interactions within the polymer matrix [44], and enables PNIPAM to respond sharply and reversibly to thermal stimuli. The integration of PNIPAM into microfluidic platforms confers unique opportunities for dynamic, programmable actuation. PNIPAM‐based hydrogel actuators have been employed as thermally controlled valves, pumps, and flow regulators [45]. This responsiveness is particularly valuable in the context of platforms equipped with individually addressable microheater arrays, which permit precise spatial and temporal control over thermal fields and, consequently, the microfluidic actuation events [46, 47]. PNIPAM hydrogels are also biocompatible [48, 49]. The operational temperature window (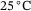 to 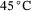) encompasses the LCST of PNIPAM and is chosen to ensure robust and reproducible performance.

The temperature‐ and time‐dependent response of the PNIPAM structures is first characterized in Figure S10. The structures exhibit a pronounced and reversible change in size across the transition temperature, with repeated heating and cooling cycles demonstrating reproducible thermal actuation. The integration of these PNIPAM structures with the programmable microheater array in a microfluidic device is demonstrated in Figure S11. Selective heating from 25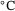 to 45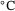 induces controlled structural deformation and spatial rearrangement, demonstrating programmable thermal manipulation of the PNIPAM structures.

While the demonstrated 1024‐element microheater array exhibits robust spatiotemporal thermal modulation capabilities, several avenues exist for further enhancing its thermal performance and scalability. Future improvements may focus on reducing the thermal time constant of individual heater elements through optimization of the heater geometry, minimization of thermal mass, and incorporation of thermally isolated microstructures. The implementation of suspended membrane architectures or low‐thermal‐conductivity support layers could substantially reduce heat dissipation into the substrate, thereby enabling faster heating and cooling dynamics while improving thermal confinement. Furthermore, mitigation of lateral heat diffusion through engineered thermal barriers or trench‐based isolation strategies would enhance spatial resolution and reduce thermal cross‐talk between adjacent pixels. From an electronic perspective, increasing the multiplexing frequency and integrating application‐specific driver circuitry could improve refresh rates and facilitate more rapid regulation by utilizing either resistance‐based thermometry or real‐time feedback from an infrared camera. Feedback would provide enhanced temperature stability, uniformity, and compensation for fabrication‐induced variations across the array. In addition, the exploration of alternative heater materials with favourable electrothermal properties may enable improved power efficiency and dynamic response.

At the system level, the integration of advanced control methodologies, including model‐based thermal compensation and data‐driven optimization algorithms, may further improve the accuracy of spatiotemporal temperature pattern generation. Collectively, these developments have the potential to significantly increase thermal response speed, spatial precision, and operational scalability, thereby expanding the applicability of programmable microheater arrays in emerging fields such as microfluidics, bio interfaces, soft robotics, adaptive materials, and high‐throughput thermal manipulation.

## 7 Conclusions

We have demonstrated the fabrication, operation and control of, what to the best of our knowledge is the largest microheater array to date. Our array consists of 1024 elements, arranged in a 32×32 closely spaced grid, where each heater has a dimension of 297.5μm. Individual elements can be heated up to 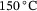 without layer delamination (see Figure S6 for further details). A double spiral‐like heater structure from Pt is used to provide a uniform temperature distribution in each heating element. The wiring challenge is solved by implementing row/column addressing. The control is achieved by a fully programmable high voltage multiplexer board that operates with a maximum refresh time of 3s which corresponds to the time needed to activate all the 1024 elements of the array and maintain them at 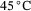. The temperature of individual microheater elements is controlled by pulsating the drive voltage and thereby regulating the total current used for Joule heating. Our microheater can be used to generate dynamic heat patterns and we demonstrate the heat‐driven melting of Gallium to obtain solid metal shapes. We believe that the scalable architecture demonstrated herein lends itself for applications that require a large number of individually addressable microheaters, as is of interest for applications that benefit from fine spatial temperature control.

## 8 Methods

### 8.1 Numerical Simulation

The spatially selective heating was simulated in MATLAB using an inbuilt finite element model that calculates the variation of heat flux and temperature along the boundaries as a function of time. The values for the thermal conductivity, mass density, and specific heat capacity for Ga are, respectively, as follows: $36.5\;Wm^{-1}\;K^{-1}$, $5910\;kgm^{-3}$, and $371\;Jkg^{-1}\;K^{-1}$. All other material properties were adopted from the built‐in library.

### 8.2 Fabrication of the Heater Array

The SiO_2_/Si substrate was purchased from Siegert Wafer GmbH. The P‐doped (Boron) silicon wafer had a 300nm SiO_2_ thermal oxide layer. First, the heater elements were fabricated by UV photolithography (Suss, MA6 mask aligner), followed by electron‐beam evaporation of Pt and a lift‐off process. This was followed by the deposition of the Ti/Au electrode/wire patterns by the same method. The isolation of the conductor lines at the crossover junctions involved the steps of UV photolithography, followed by the electron‐beam deposition of 1μm HfO_2_. The Ti/Au layers were sputtered sequentially by an electron‐beam evaporator (Thermionics). Finally, the device was coated with a parylene layer (PDS 2010, Specialty Coating Systems GmbH) via chemical vapor deposition using a two temperature step program (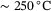 and 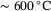). The layer thickness was determined by the amount of loaded parylene dimers (precursor) and was estimated to be around 1μm. The Ti, Au, and HfO_2_ material for electron‐beam evaporator system were purchased from Kurt J. Lesker GmbH. The different layers of the fabrication process are graphically presented in Figures S2–S5.

### 8.3 Architecture of the Microheater Array

The row and column electrodes of 32×32 heater array are connected to a custom‐built digitally controlled electronics board to enable individual addressing of the microheater elements. The primary function of the board is to control the high voltage (*V*_DD_) channel switch with a low voltage digital signal (*V*_LL_). The high‐voltage switch opens or closes the circuit to generate the target pattern. The switch is normally open and requires a 5‐bit code for switching operation which is controlled by a microcontroller (Arduino Mega 2560). The row electrodes are connected to the output terminal of a high voltage amplifier (WMA‐300, Falco Systems GmbH) and the column electrodes are connected to ground. The following electronics components are used in the construction of the board: Demultiplexer ‐ CD74HC4067M (Texas Instruments), Shift‐Register ‐ SNLS674 (Texas Instruments), and High Voltage Channel Switch ‐ TMUX9616 (Texas Instruments). LPC 6/3‐ST‐7.62 PCB connectors (Phoenix Contact) are soldered on the board for the DC and HV voltage supply. The thermal pattern is generated by sequentially switching ON the microheater pixels where the 5‐bit code is loaded through external CLK pulses. The pixels are heated for a fixed duration based on the temperature requirements of the output. The high voltage signal profile remains unchanged, but the switches are controlled via the 5‐bit input code and the CLK pulses.

### 8.4 Digital Signal Processing to Address Heater Elements

Individual heating elements are addressed via the 32 bus lines and an input wordline of total 5 bits. The first bit of the word line represents the chip‐select (CS) bit and the remaining bits are WL[i], WL[j], WL[k], and WL[l]. The digital circuit selects the corresponding row/column based on the five input bits, and triggers the high voltage switch (HVS) such that the voltage $V_{DD}$ and ground signals connect to the selected row and column bus lines, respectively. The schematic representation of the electrical architecture is shown in Figure 3A.

The bit‐transfer is exercised through external clock (CLK) as shown in the Figure 3A. An image of the device's digital circuit board is shown in Figure 3B, where different integrated components and input/output lines are highlighted with color‐dotted lines. The circuit board controls the switching of the 32×32 microheater array with a five‐input logic provided though input control lines. The OFF state of the HVS represents the floating ground signal and the ON state represents $V_{DD}$ and ground signals to R and C respectively. The selected cell will allow current to flow through the microheater element and accordingly generate the thermal response. The serial input to the high voltage channel switch (HVS) connects the voltage signal $V_{DD}$ to the respective buslines (R/C) for the selective operation of microheater pixel. The busline index (R/C) as a function of wordline can be written as $2^{WL[i]}+2^{WL[j]}+2^{WL[k]}+2^{WL[l]}$ where “ijkl” is the four bit wordline. Multiple microheater elements are addressed sequentially using time‐division multiplexing of the clock signal. The array is also equipped with a driving voltage line and a ground line. The selected row and column cells are in accordance with the output addresses sent from the parallel‐in/ serial‐out shift register (PI/SO SR).

### 8.5 Infrared Thermography

Thermal characterization of the microheater array was performed using a FLIR A35 infrared camera. Because the heater elements were encapsulated beneath a parylene passivation layer, an emissivity value of 0.96 was used for all thermal measurements, corresponding to the exposed parylene‐coated device surface. To validate the infrared temperature measurements, a K‐type thermocouple was placed in direct thermal contact with the device surface and used as an independent temperature reference. Temperature measurements were acquired simultaneously using infrared thermography and thermocouple sensing under identical heating conditions. The emissivity setting was verified through comparison of the two measurement techniques, and the resulting calibration was applied to all thermal imaging experiments. The calibrated infrared temperatures exhibited a maximum deviation of less than 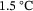 from the thermocouple measurements over the investigated temperature range, confirming the accuracy of the thermal characterization presented in this work.

## Conflicts of Interest

The authors declare no conflicts of interest.

## Supporting information


**Supporting File 1**: advs77563‐sup‐0001‐SuppMat.pdf.

**Supporting File 2**: advs77563‐sup‐0002‐VideoS1.mp4.

**Supporting File 3**: advs77563‐sup‐0003‐VideoS2.mp4.

**Supporting File 4**: advs77563‐sup‐0004‐VideoS3.mp4.

**Supporting File 5**: advs77563‐sup‐0002‐VideoS1.mp4.

## Data Availability

The data that support the findings of this study are available from the corresponding author upon reasonable request.
