# Supplementary material for: Spatiotemporal Thermal Modulation and Patterning using a Programmable 1024 Element Microheater Array
Source: Adv Sci (Weinh). 2026 Sep 11:e77563. Online ahead of print. doi: 10.1002/advs.77563 (PMC13564193; doi:10.1002/advs.77563)
Supplement: Supplementary file 1 [file ADVS-9999-e77563-s005.pdf]

Supplementary Information

Spatiotemporal Thermal Modulation and Patterning using a Programmable 1024 Element Microheater Array

Rahul Goyal, Jang-Hwan Han, Sadaf Pashapour, and Peer Fischer

CONTENTS

Supplementary Note 1: Power calculations for microheater array **2**

Supplementary Note 2: Device Operation **3**

Supplementary Table 1: Detailed comparison of the presented microheater array with various heaters reported in previous research. **4**

Supplementary Figure 1: Thermal characteristics of microheater array under different operating conditions. **5**

Supplementary Figure 2: Fabrication process flow for 1st layer of the microheater array. **6**

Supplementary Figure 3: Fabrication process flow for 2nd layer of the microheater array. **7**

Supplementary Figure 4: Fabrication process flow for 3rd layer of the microheater array. **8**

Supplementary Figure 5: Fabrication process flow for 4th layer of the microheater array. **9**

Supplementary Figure 6: Dynamic thermal characteristics of the programmable microheater platform. **10**

Supplementary Figure 7: Electrical characteristics of the 1024-element microheater array. **11**

Supplementary Figure 8: Thermal cross-talk matrix for the 1024-element microheater array. **12**

Supplementary Figure 9: Row–column addressing architecture for scalable control of a 32 $\times$ 32 array. **13**

Supplementary Figure 10: Thermoresponsive deformation and reversible actuation of PNIPAM microstructures. **14**

Supplementary Figure 11: Thermally induced actuation of PNIPAM microstructures using the programmable microheater array. **15**

Supplementary Note 1: Power calculations for microheater array

We account for the power consumption of the microheater array by considering square wave excitation with number of pulses controlling the temperature of the microheater. A resistive microheater element with resistance R driven by an applied voltage V; the instantaneous power during the ON state is given by:

$$P_{\mathrm{ON}} = \frac{V^2}{R} \tag{S1}$$

or equivalently,

$$P_{\mathrm{ON}} = VI, \quad \text{where } I = \frac{V}{R} \tag{S2}$$

Considering square-wave excitation voltage, with period T and pulse width T_{ON}, the duty cycle D is defined as:

$$D = \frac{T_{\mathrm{ON}}}{T} \tag{S3}$$

The average power (P_A) over a cycle is given by:

$$P_{\mathrm{A}} = P_{\mathrm{ON}} \cdot D = \frac{V^2}{R} D \tag{S4}$$

The energy delivered in a single pulse (E_P) is defined by:

$$E_{\mathrm{P}} = P_{\mathrm{ON}} \cdot T_{\mathrm{ON}} = \frac{V^2}{R} T_{\mathrm{ON}} \tag{S5}$$

The total energy for excitation with N identical pulses, is given by:

$$E_{\mathrm{N}} = N \frac{V^2}{R} T_{\mathrm{ON}} \tag{S6}$$

The average power if the total experiment duration is $T_{\mathrm{T}} = NT$, becomes:

$$P_{\mathrm{A}} = \frac{E_{\mathrm{T}}}{T_{\mathrm{T}}} = \frac{V^2}{R} \frac{T_{\mathrm{ON}}}{T} = \frac{V^2}{R} D \tag{S7}$$

The ON-state power for applied voltage and resistance of 75 V and 20 mA is:

$$P_{\mathrm{ON}} = 75 \times 20 \times 10^{-3} = 1.5\,\mathrm{W} \tag{S8}$$

The time average power for multiplexed operation of the microheater array is given by:

- Case I: D = 50%

$$P_{\mathrm{A}} = 1.5 \times 0.5 = 0.75\,\mathrm{W} \tag{S9}$$

- Case II: D = 70%

$$P_{\mathrm{A}} = 1.5 \times 0.7 = 1.05\,\mathrm{W} \tag{S10}$$

The reported power value corresponds to the time-averaged electrical power consumption during row–column multiplexed operation. The sequential addressing architecture makes a single element of the microheater array active at a given time. Consequently, the reported value should not be interpreted as the power dissipated by an individual heater element or as the theoretical power consumption associated with simultaneous activation of all array elements.

Supplementary Note 2: Device Operation

We account for the power consumption of the microheater array by considering square wave excitation with number of pulses controlling the temperature of the microheater. A resistive microheater element with resistance R driven by an applied voltage V; the instantaneous power during the ON state is given by: The 32 × 32 microheater array is controlled using a custom-built digitally addressed electronics platform designed to enable individual access to each heater element while minimizing the number of external interconnects. As illustrated in Fig. S9, the row and column electrodes of the array are interfaced with a dedicated control board comprising demultiplexers, shift registers, high-voltage channel switches, and a microcontroller. The primary function of the electronics is to control a high-voltage (V_{DD}) switching network using low-voltage digital control signals (V_{LL}). The row electrodes are connected to the output of a high-voltage amplifier (WMA-300, Falco Systems GmbH), while the column electrodes are connected to ground. Individual microheater elements, denoted as (H_{ij}), are addressed through a row–column multiplexing architecture, where each heater is located at the intersection of row (R_i) and column (C_j). Activation of a heater occurs only when both the corresponding row and column channels are selected, satisfying the logical condition ($H_{ij} = R_i C_j$).

Address selection is performed using a 5-bit digital code generated by an Arduino Mega 2560 microcontroller and decoded by CD74HC4067M demultiplexers (Texas Instruments). The switching states are synchronized through external clock (CLK) pulses and managed using SNLS674 shift registers (Texas Instruments), which serially load the addressing information into the switching circuitry. High-voltage routing is accomplished using TMUX9616 high-voltage channel switches (Texas Instruments), which remain normally open and close only when the appropriate 5-bit address is received. In this configuration, the microcontroller sequentially loads the address code corresponding to the target row and column, and the selected high-voltage switch completes the electrical path between the amplifier output and the desired microheater element. LPC 6/3-ST-7.62 PCB connectors (Phoenix Contact) are incorporated on the control board to provide reliable high-voltage and DC power connections.

Thermal patterns are generated through rapid sequential activation of individual microheater pixels. For each selected pixel, the corresponding 5-bit address is loaded into the control circuitry via CLK pulses, causing the associated high-voltage switch to close while all non-selected channels remain electrically isolated. The selected heater is energized for a predetermined duration that is defined by the desired local temperature distribution. Throughout operation, the amplitude and temporal profile of the applied high-voltage signal remain unchanged; instead, spatial pattern generation is achieved exclusively through digital control of the switching network. By continuously scanning and updating the row–column addresses, arbitrary thermal patterns can be produced across the entire 32 × 32 array while substantially reducing wiring complexity and enabling straightforward scalability to larger arrays.

**Material**	**Maximum Temperature ($\mathbf{T_M}$)**	**Complex Thermal Output**	**Pixel Area**	**Number of Pixels ($\mathbf{N \times N}$)**	**Applied Power ($\mathbf{P_M}$)**
Pt [1]	498 °C	ND	2.3 mm × 2.3 mm	3×3	281 mW × N^2
Poly-Si [2]	350 °C	ND	1.1 mm × 1.1 mm	1×1	175 mW × N^2
Pt–Ti [3]	80 °C	ND	0.6 mm × 0.6 mm	4×4	NR
Cr [4]	NR	ND	0.2 mm × 0.2 mm	4×4	NR
PVC-Gel [5]	60 °C	Row/Column Addressable Electrodes	10 mm × 10 mm	5×5	1.05 W × N^2
CNTs [6]	100 °C	Individually Addressable Electrodes	0.9 mm × 0.9 mm	16×16	160 mW × N^2
Expanded Intercalated Graphite [7]	100 °C	Individually Addressable Electrodes	10 mm × 10 mm	6×6	1.8 W × N^2
Pt [Current work]	150 °C	Row/Column Addressable Electrodes	0.25 mm × 0.25 mm	32×32	0.87 W

Supplementary Table S1: Detailed comparison of the presented microheater array with various heaters reported in previous research.

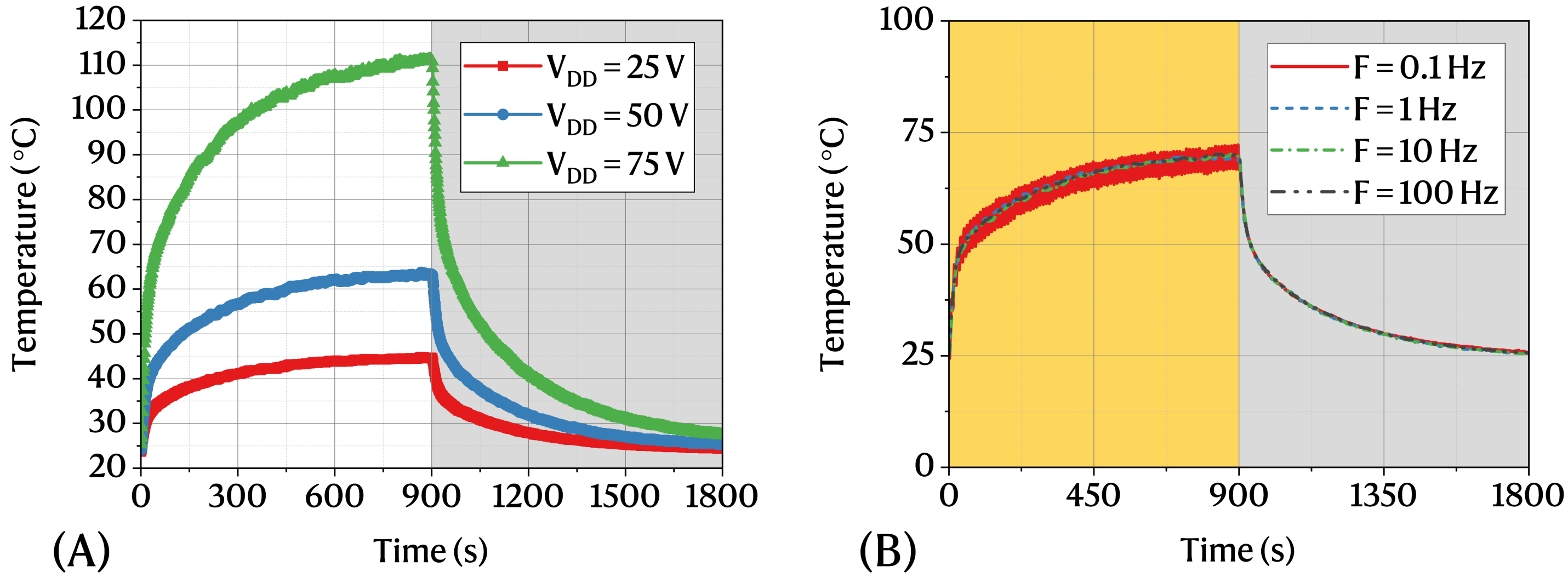

Supplementary Figure S1: **Thermal characteristics of microheater array under different operating conditions.** (A) The effect of DC voltage (V_{DD}) on the heating and cooling performance after 900 sec of continuous operation. (iii) The effect of the frequency of the applied AC signal on the heating and cooling performance of the element under 75 V AC square wave signal.

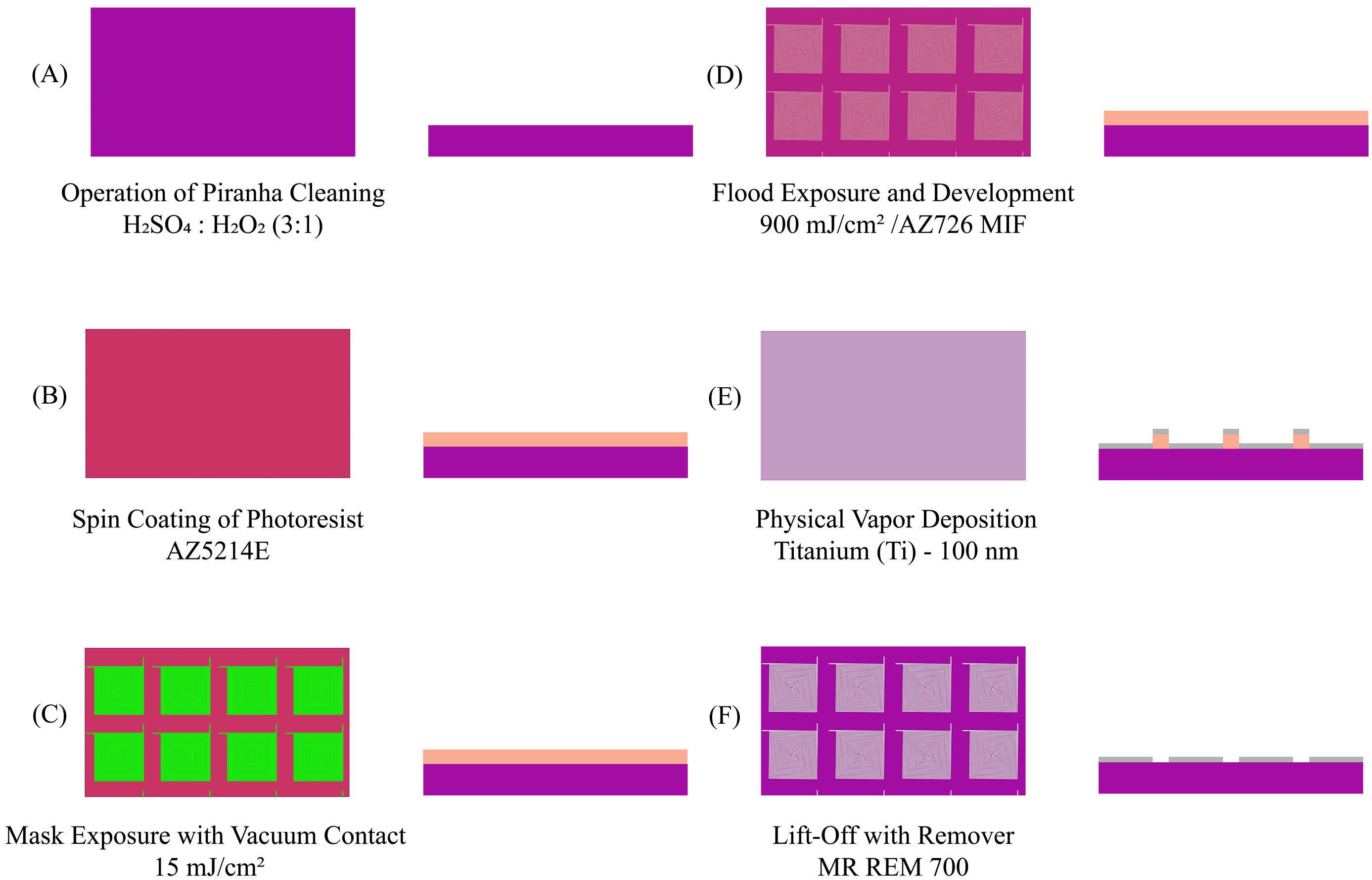

Supplementary Figure S2: **Fabrication process flow for 1st layer of the microheater array.** Left side of the panel shows the top view and right side represents the side view of the device fabrication stage.

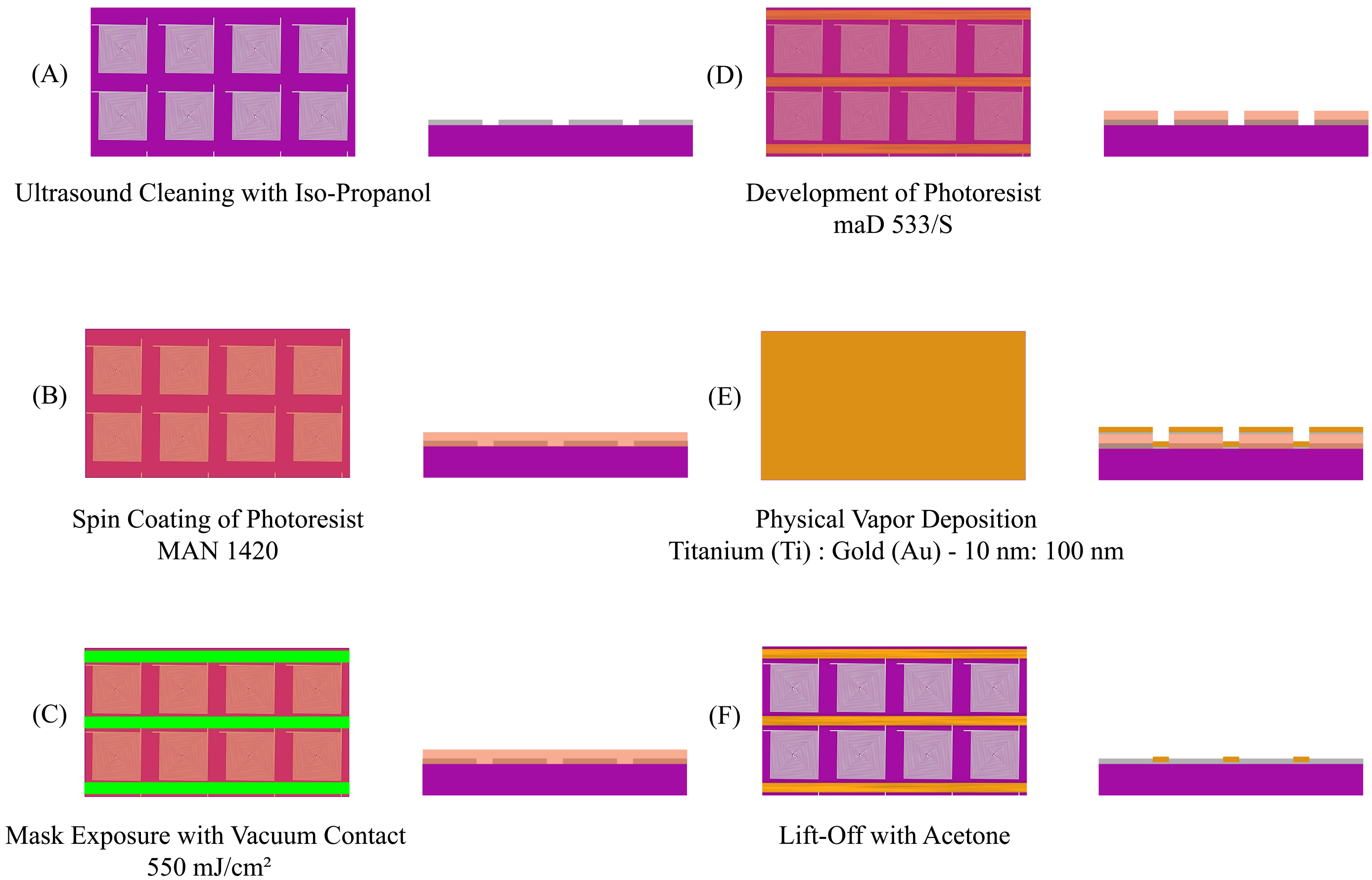

Supplementary Figure S3: **Fabrication process flow for 2nd layer of the microheater array.** Left side of the panel shows the top view and right side represents the side view of the device fabrication stage.

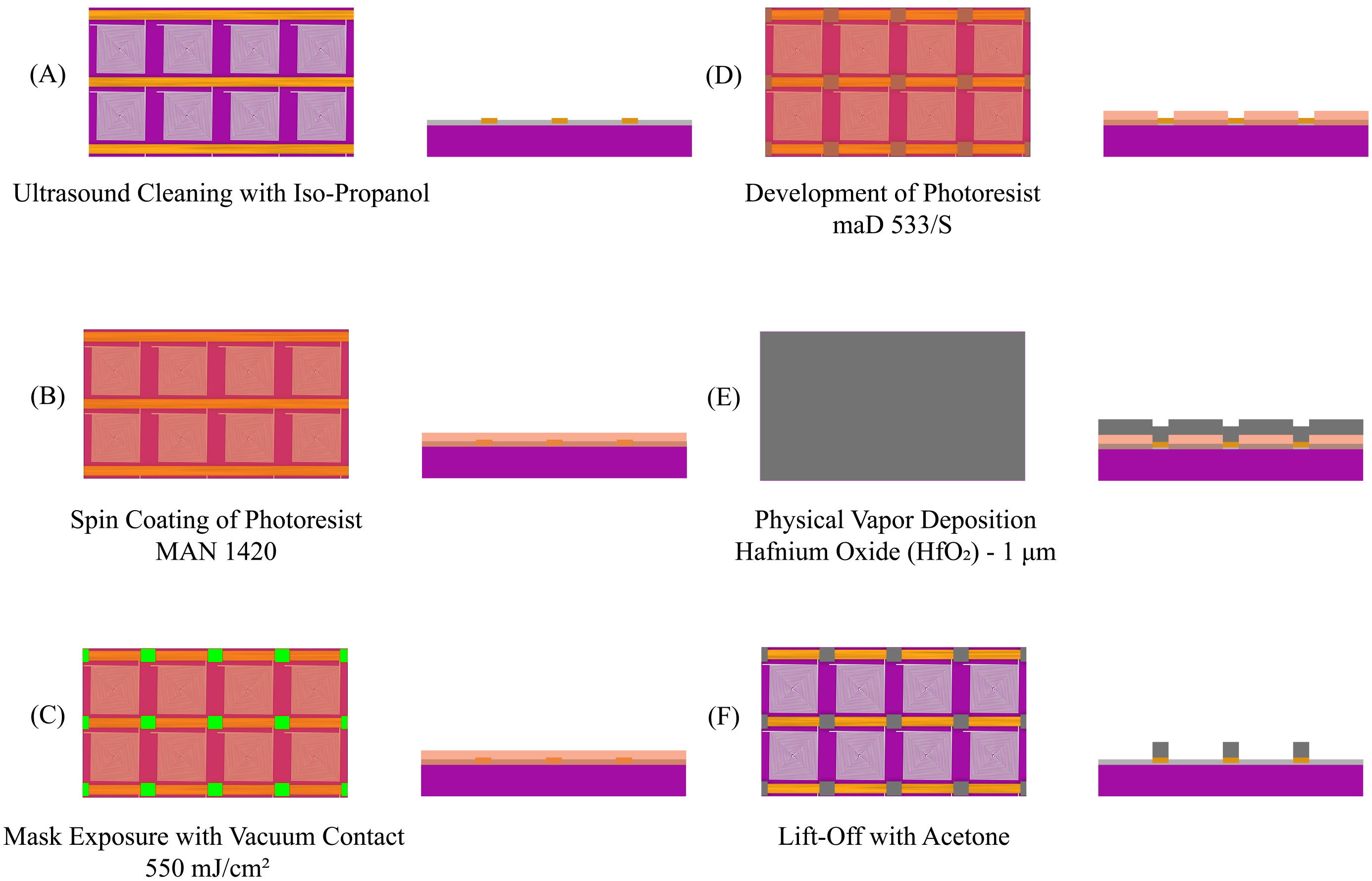

Supplementary Figure S4: **Fabrication process flow for 3rd layer of the microheater array.** Left side of the panel shows the top view and right side represents the side view of the device fabrication stage.

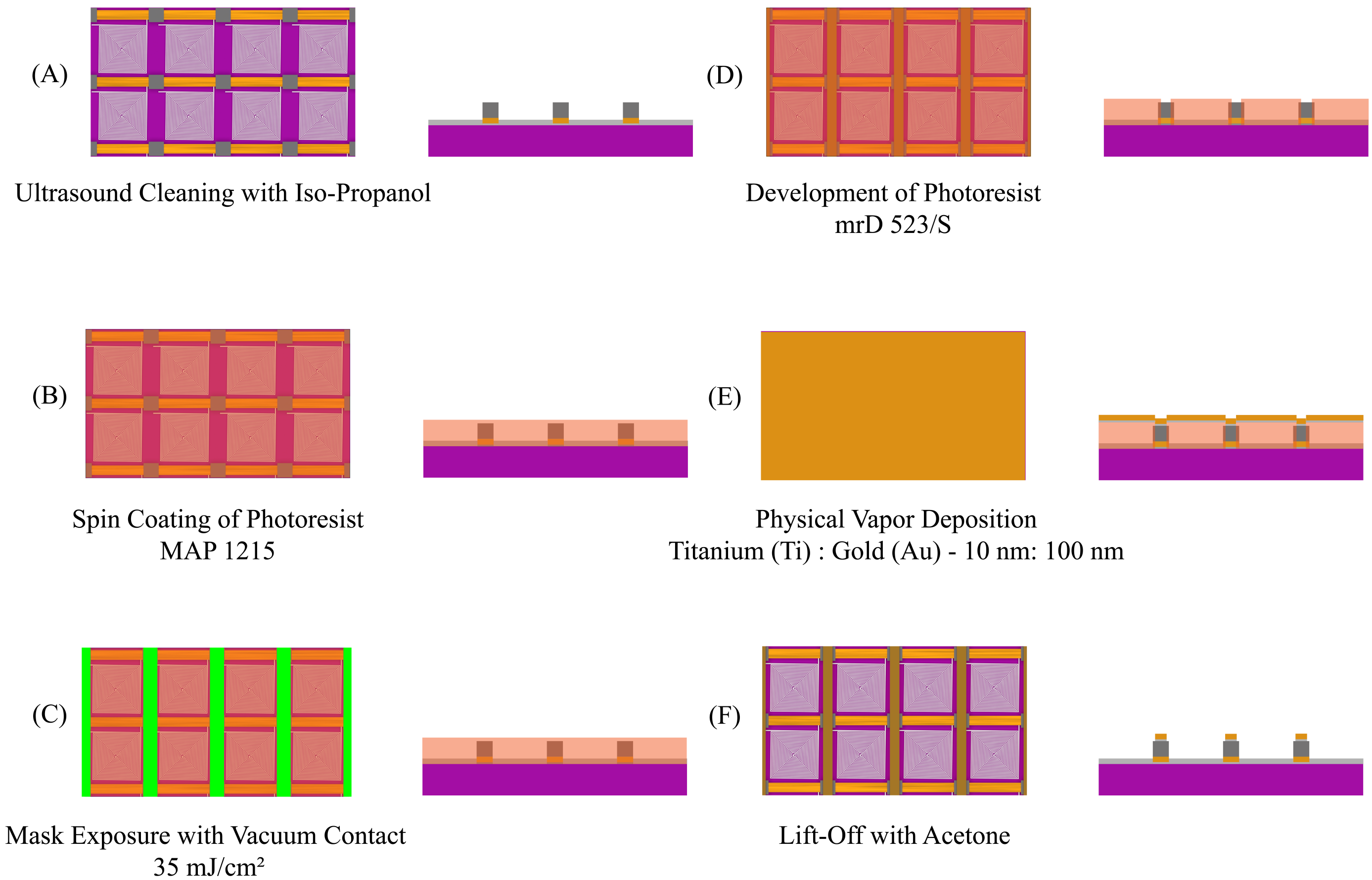

Supplementary Figure S5: **Fabrication process flow for 4th layer of the microheater array.** Left side of the panel shows the top view and right side represents the side view of the device fabrication stage.

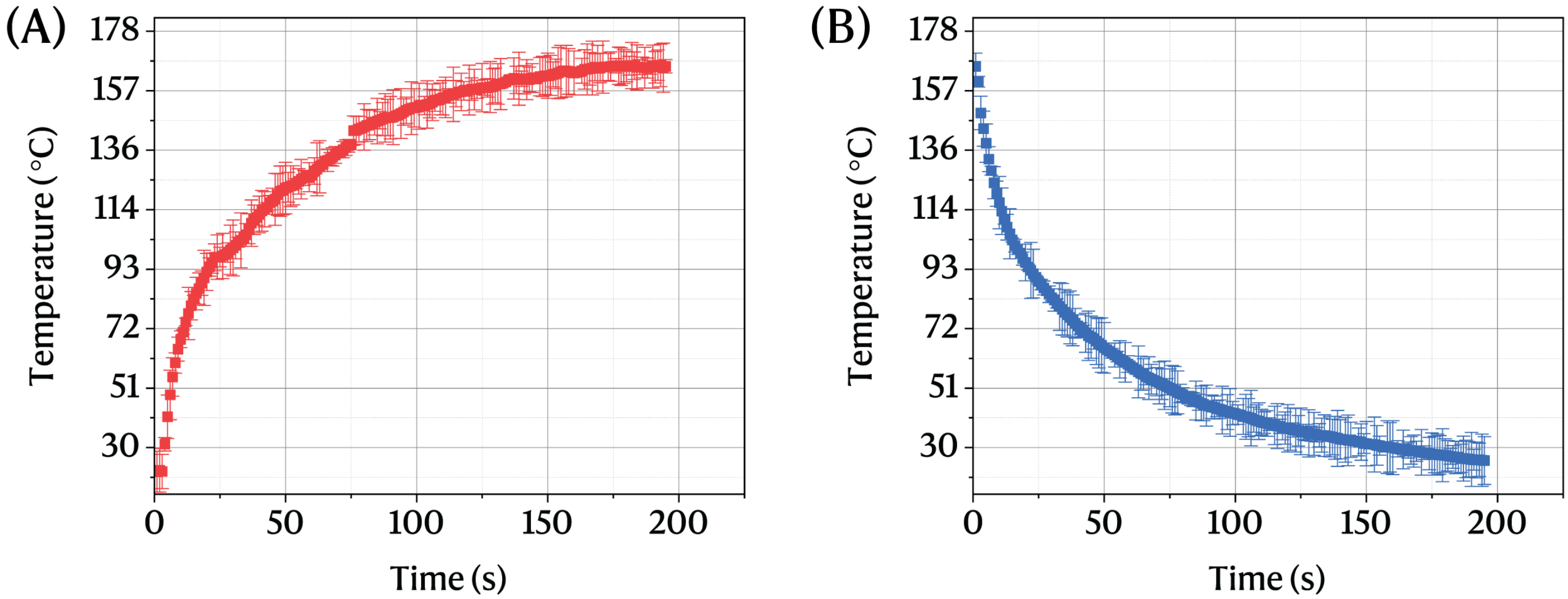

Supplementary Figure S6: **Dynamic thermal characteristics of the programmable microheater platform.** (A) Temperature evolution during Joule heating of the fabricated platinum microheater. (B) Temperature decay following termination of the heating stimulus. The device achieves temperatures exceeding 160 °C while maintaining reproducible thermal behaviour over repeated measurements. Analysis of the transient response yields a heating rise time (10–90%) of ≈ 95 sec and a cooling fall time (90–10%) of ≈ 108 sec, demonstrating stable and reversible thermal actuation suitable for programmable spatiotemporal temperature modulation.

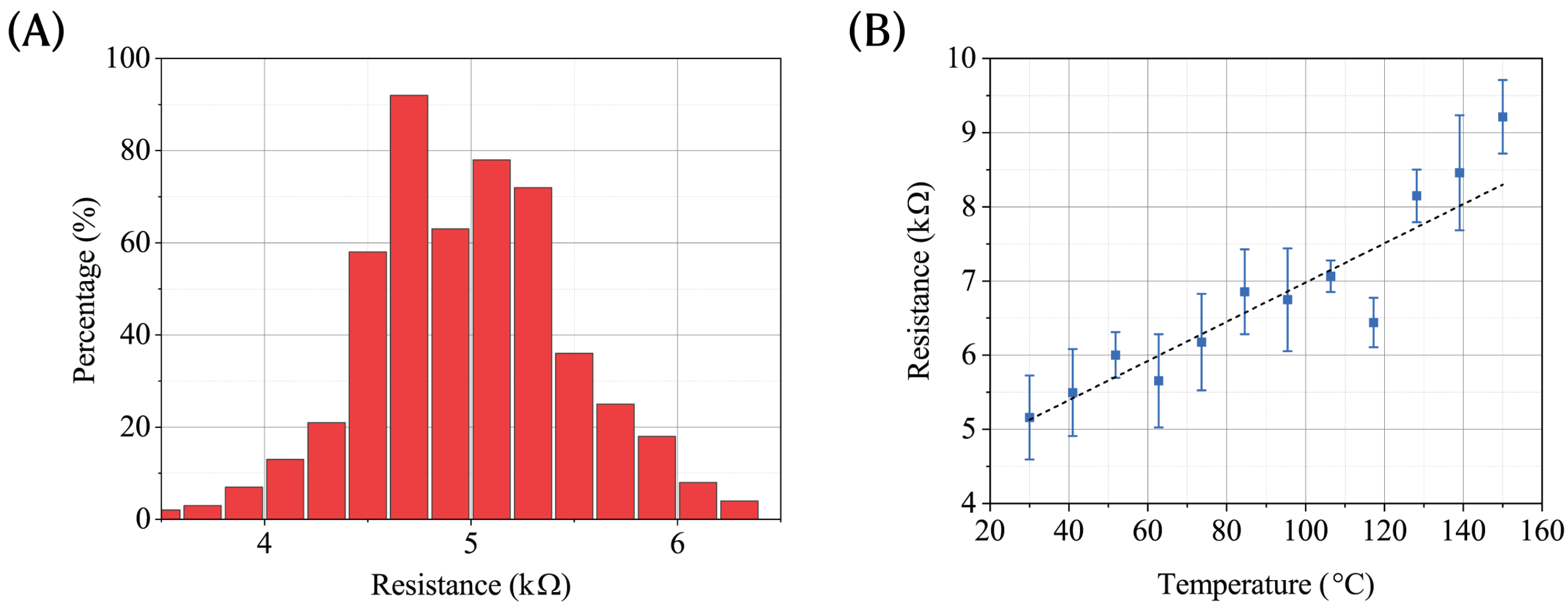

Supplementary Figure S7: **Electrical characteristics of the 1024-element microheater array.** (A) Statistical distribution of the room-temperature resistance of individual platinum microheaters, highlighting the high degree of device-to-device uniformity achieved through microfabrication. (B) Resistance as a function of temperature for a representative heater element, exhibiting a linear thermoresistive response across the investigated temperature range. This behaviour enables reliable electrical temperature calibration and provides a foundation for precise spatiotemporal thermal control within the array.

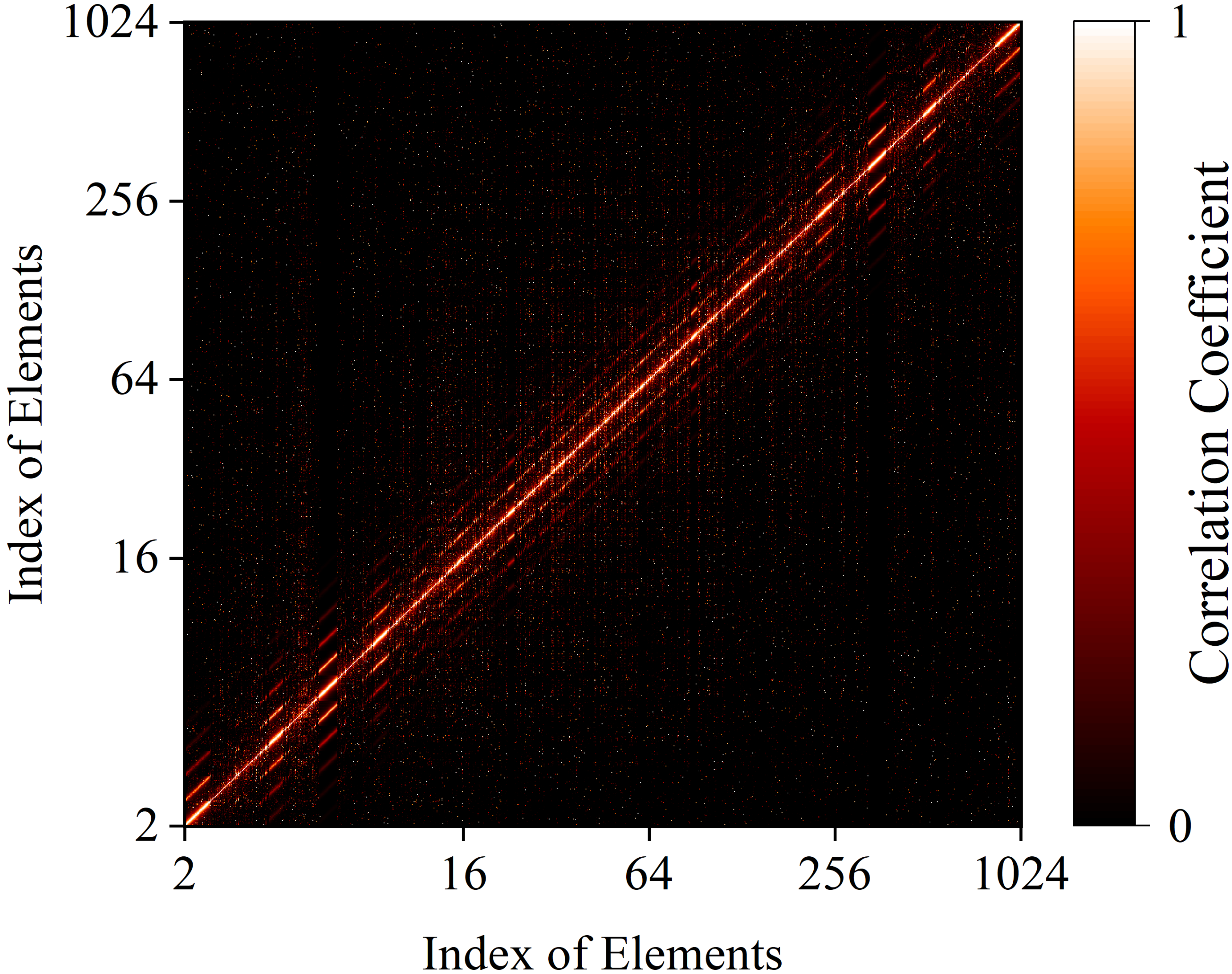

Supplementary Figure S8: **Thermal cross-talk matrix for the 1024-element microheater array.** Pairwise correlation matrix of the thermal responses measured from all 1024 individually addressable microheater elements. The rapid attenuation of the off-diagonal correlations indicates that thermal interactions are predominantly confined to neighbouring elements.

(A)

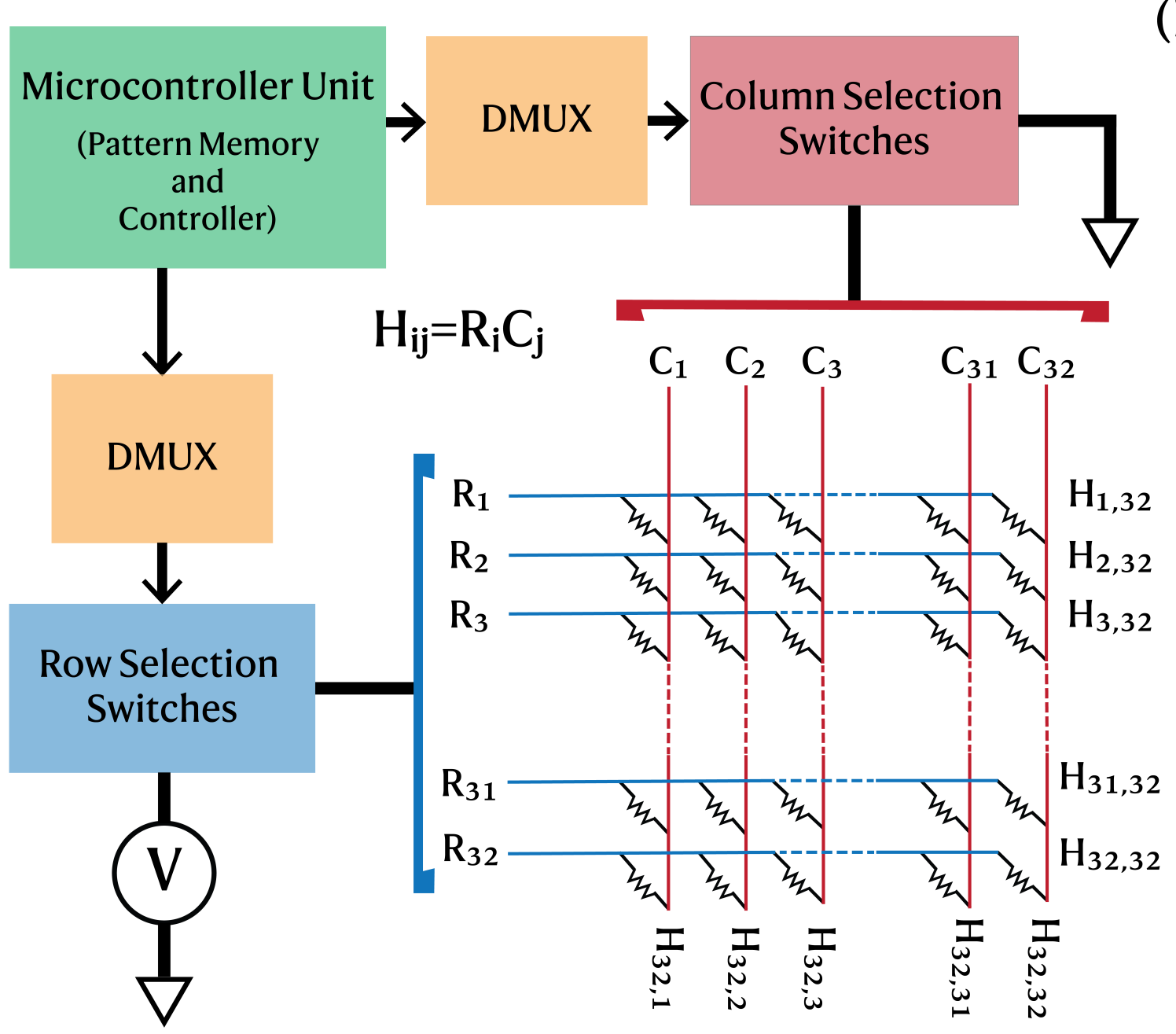

Row Signal, R_i	Column Signal, C_j	Element State, H_{ij}
0	0	OFF
0	1	OFF
1	0	OFF
1	1	ON

(B)

ADDRESS	ACTIVE ROW	ACTIVE COLUMN	SELECTED ELEMENT
00000	R_1	C_1	$H_{1,1}$
00001	R_2	C_2	$H_{2,2}$
....	....	....	....
01111	R_{16}	C_{16}	$H_{16,16}$
10000	R_{17}	C_{17}	$H_{17,17}$
....	....	....	....
11110	R_{31}	C_{31}	$H_{31,31}$
11111	R_{32}	C_{32}	$H_{32,32}$

Supplementary Figure S9: **Row–column addressing architecture for scalable control of a 32 $\times$ 32 array.** (A) Schematic of the addressing system comprising a microcontroller unit (MCU), demultiplexers (DMUXs), and row- and column-selection switches. Individual elements (H_{ij}) are addressed at the intersection of row (R_i) and column (C_j), following the logic relation ($H_{ij} = R_iC_j$). The truth table shows that an element is activated only when both the corresponding row and column signals are high. (B) Binary addressing scheme used to sequentially select row–column pairs within the array. Each 5-bit address uniquely maps to a row and column, enabling access to the corresponding element, from ($H_{1,1}$) to ($H_{32,32}$).

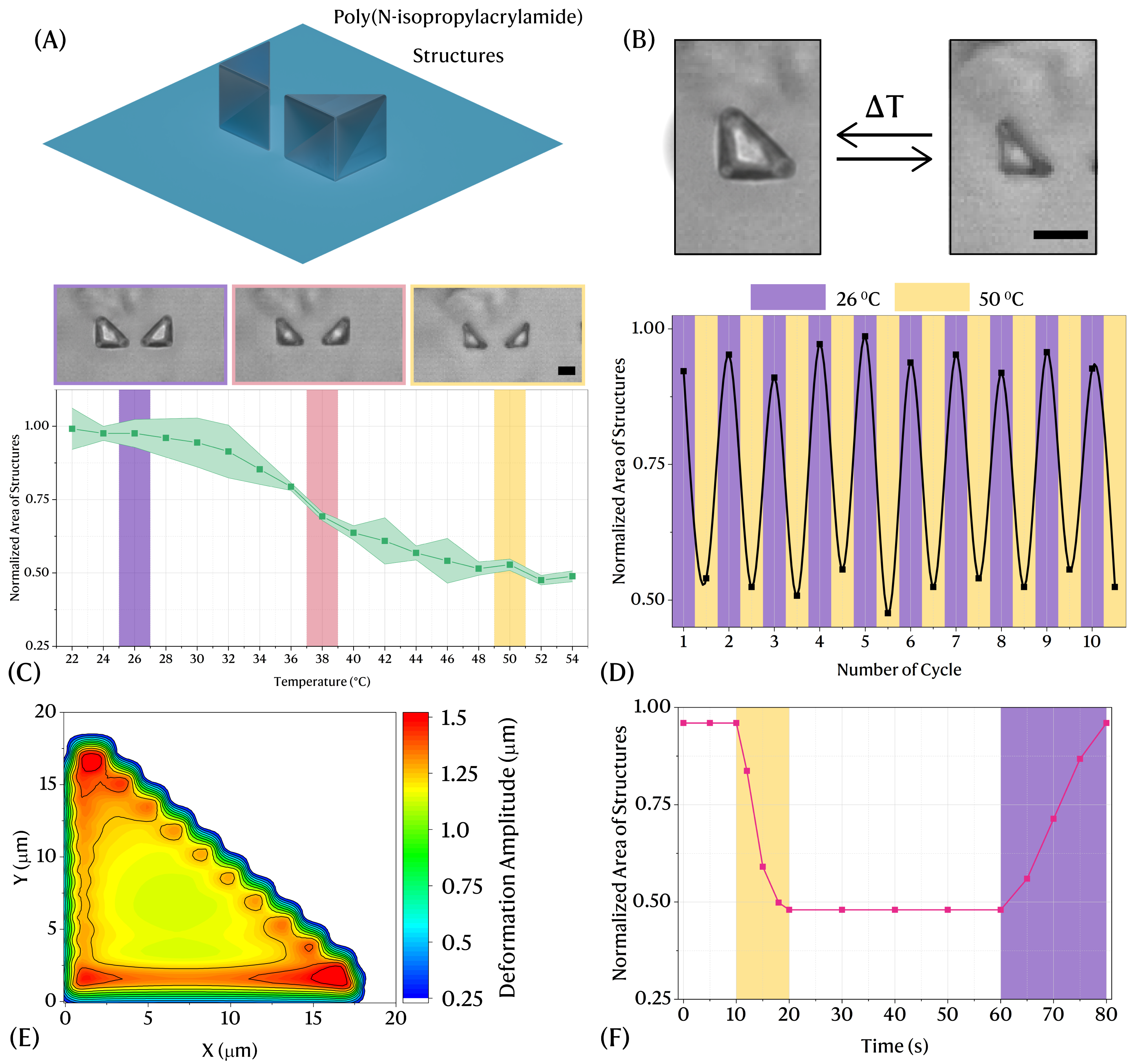

Supplementary Figure S10: **Thermoresponsive deformation and reversible actuation of PNIPAM microstructures.** (A) Schematic illustration of two microstructures on a substrate, employed for temperature-responsive deformation. (B) Representative optical microscopy images of individual microstructures in their expanded and contracted states; the scale bar indicates the length of 10 µm. (C) Temperature-dependent change in the normalized area of the structures, showing a gradual decrease in area with increasing temperature. The shaded regions indicate selected temperature ranges; the scale bar indicates the length of 5 µm (D) Reversible change in normalized structure area over ten heating–cooling cycles, demonstrating repeatable thermoresponsive actuation. (E) Representative spatial distribution/-contour map of the deformation profile of the PNIPAM microstructure. (F) Time-dependent response of the normalized structure area during heating and cooling, showing rapid contraction upon heating, a stable contracted state, and recovery toward the initial state upon cooling (average response time $< 15\,\mathrm{s}$).

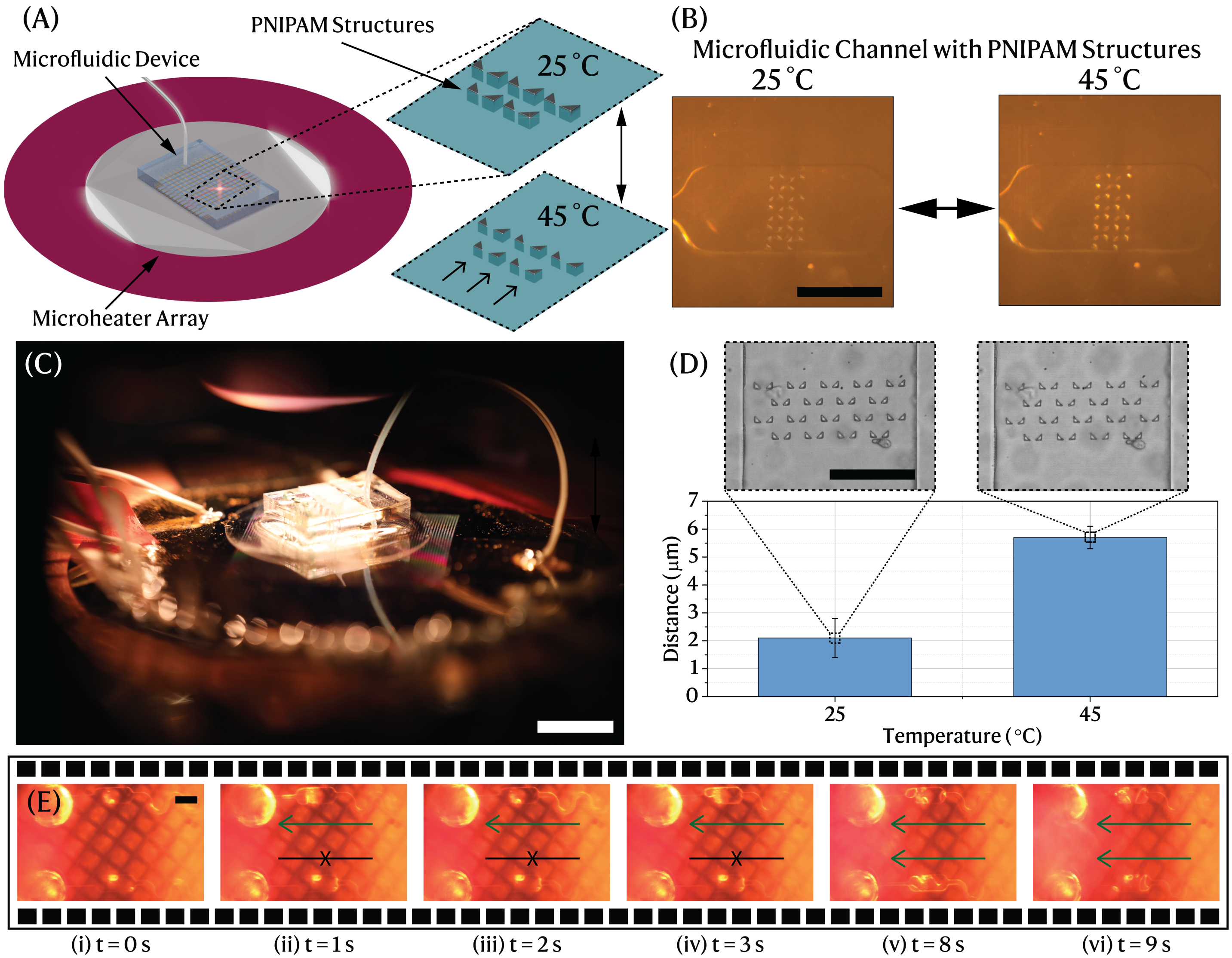

Supplementary Figure S11: **Thermally induced actuation of PNIPAM microstructures using the programmable microheater array.** (A) Schematic illustration of the experimental platform consisting of a microfluidic device integrated with the programmable microheater array. Temperature modulation induces a reversible volume-phase transition of the PNIPAM microstructures, resulting in temperature-dependent shape changes between 25 °C and 45 °C. (B) Optical micrographs of PNIPAM structures within the microfluidic channel at 25 °C and 45 °C, demonstrating reversible thermoresponsive actuation upon localized heating. (C) Photograph of the assembled microfluidic device mounted on the microheater array during operation. (D) Quantification of the thermally induced displacement of the PNIPAM structures at 25 °C and 45 °C. Insets show representative optical images used for displacement analysis. Error bars represent the standard deviation from multiple measurements. (E) Time-lapse thermal imaging sequence illustrating dynamic actuation of the PNIPAM structures under programmable thermal stimulation. Arrows indicate the direction of continuous fluid motion, while the cross denotes the restricted fluid motion. The sequence demonstrates selective and reversible control of the microfluidics channels thermally driven deformation enabled by spatiotemporal temperature control of the microheater array.

REFERENCES

[1] Y. Bai, J. Tian, Z. Lin, M. You, J. Liu, X. Wang, *Microsystem Technologies* **2020**, *26* 787.

[2] W.-J. Hwang, K.-S. Shin, J.-H. Roh, D.-S. Lee, S.-H. Choa, *Sensors* **2011**, *11* 2580.

[3] W. Jung, Y. W. Kim, D. Yim, J. Y. Yoo, *Sensors and Actuators A: Physical* **2011**, *171* 228.

[4] M. Horade, M. Kojima, K. Kamiyama, Y. Mae, T. Arai, *Mechanical Engineering Research* **2016**, *6* 66.

[5] M. Kim, M. Cho, C. Chung, K.-U. Kyung, *npj Flexible Electronics* **2024**, *8* 59.

[6] P. Liu, L. Liu, K. Jiang, S. Fan, *Small* **2011**, *7* 732.

[7] R. A. Bilodeau, M. C. Yuen, R. Kramer-Bottiglio, *Advanced Materials Technologies* **2019**, *4*.
